# Transcriptome data of control and *Ascosphaera apis* infected *Apis mellifera ligustica* larval guts

**DOI:** 10.1016/j.dib.2020.105264

**Published:** 2020-02-08

**Authors:** Huazhi Chen, Yu Du, Zhiwei Zhu, Cuiling Xiong, Yanzhen Zheng, Dafu Chen, Rui Guo

**Affiliations:** College of Animal Sciences (College of Bee Science), Fujian Agriculture and Forestry University, Fuzhou 350002, China

**Keywords:** Western honeybee, *Apis mellifera ligustica*, *Ascosphaera apis*, Larvae, Gut, Transcriptome

## Abstract

*Ascosphaera apis* is an obligate fungal pathogen of honeybee larvae that leads to chalkbrood, which causes heavy losses for the apiculture in China and many other countries. In this article, guts of 4-, 5-, 6-day-old *Apis mellifera ligustica* larvae challenged by *A. apis* (AmT1, AmT2, AmT3) and normal 4-day-old larval guts (AmCK) were sequenced using next-generation sequencing technology. On average, 29,196,197, 28,690,943, 29,779,715 and 30,496,725 raw reads were yielded from these four groups; an average of 29,540,895 clean reads were obtained after quality control. In addition, the mapping ratio of clean reads in treatment and control groups to the *Apis mellifera* genome were over 97.16%. For more insight please see “Uncovering the immune responses of *Apis mellifera ligustica* larval gut to *Ascosphaera apis* infection utilizing transcriptome sequencing” [1]. The raw data were submitted to the National Centre for Biotechnology Information (NCBI) Sequence Read Archive (SRA) database under accession numbers: SRR4084091, SRR4084092, SRR4084095, SRR4084096, SRR4084097, SRR4084098, SRR4084099, SRR4084100, SRR4084101, SRR4084102, SRR4084093, SRR4084094.

**Table:** Specifications Table

Subject	Immunology and Microbiology (General)
Specific subject area	Transcriptomics
Type of data	Table
How data were acquired	Illumina HiSeq™ 2500
Data format	Raw sequences (FASTQ)
Parameters for data collection	Uninfected and *Ascosphaera apis*-infected larval guts of *Apis mellifera ligustica* were harvested and stored in liquid nitrogen, followed by total RNA isolation, cDNA library construction and high-throughput sequencing on an illumina HiSeq™ 2500 platform using paired-end strategy.
Description of data collection	Guts in treatment groups were sampled from 4-, 5-, 6-day-old *A. m. ligustica* larvae infected with *A. apis* (AmT1, AmT2, AmT3), while guts in control group were sampled from 4-day-old *A. m. ligustica* larvae (AmCK). Total RNA of control and treatment groups were isolated followed by cDNA library construction and next-generation sequencing.
Data source location	College of Animal Sciences (College of Bee Science), Fujian Agriculture and Forestry University, Fuzhou, China (latitude 26° 5′ 14.9856″N and longitude 119° 14′ 2.8896″W)
Data accessibility	Repository name: National Centre for Biotechnology Information (NCBI) Sequence Read Archive (SRA) database Data identification numbers: SRR4084091, SRR4084092, SRR4084095, SRR4084096, SRR4084097, SRR4084098, SRR4084099, SRR4084100, SRR4084101, SRR4084102, SRR4084093, SRR4084094. Direct URL to data: https://www.ncbi.nlm.nih.gov/sra/?term=SRR4084091, https://www.ncbi.nlm.nih.gov/sra/?term=SRR4084092, https://www.ncbi.nlm.nih.gov/sra/?term=SRR4084095, https://www.ncbi.nlm.nih.gov/sra/?term=SRR4084096, https://www.ncbi.nlm.nih.gov/sra/?term=SRR4084097, https://www.ncbi.nlm.nih.gov/sra/?term=SRR4084098, https://www.ncbi.nlm.nih.gov/sra/?term=SRR4084099, https://www.ncbi.nlm.nih.gov/sra/?term=SRR4084100, https://www.ncbi.nlm.nih.gov/sra/?term=SRR4084101, https://www.ncbi.nlm.nih.gov/sra/?term=SRR4084102, https://www.ncbi.nlm.nih.gov/sra/?term=SRR4084093, https://www.ncbi.nlm.nih.gov/sra/?term=SRR4084094.
Related research article	D.F. Chen, R. Guo, X.J. Xu, C.L. Xiong, Q. Liang, Y.Z. Zheng, Q. Luo, Z.N. Zhang, Z.J. Huang, D. Kumar, W. Xi, X. Zou, M. Liu. Uncovering the immune responses of *Apis mellifera* ligustica larval gut to *Ascosphaera apis* infection utilizing transcriptome sequencing. Gene 621 (2017) 40–50 [1].

**Table:** 

**Value of the data** • This transcriptome data provides comprehensive information about mRNAs in control and *A. apis*-infected *A. m. ligustica* larval guts. • The data contribute in understanding interactions between *A. m. ligustica* larvae and *A. apis* during chalkbrood. • Current data can benefit understanding pathways and genes involved in *A. m. ligustica* response to *A. apis* infection.

## 1 Data description

On average, 29,196,197, 28,690,943, 29,779,715 and 30,496,725 raw reads were respectively generated from AmT1, AmT2, AmT3 and AmCK (Table 1). After strict quality control, an average of 29,540,895 clean reads were obtained, and the ratio of clean reads were over 97.16% in each group (Table 1). Additionally, the mapping ratio of clean reads in each group to the reference genome of *A. mellifera* were ranged from 86.25% to 89.45% (Table 2). The raw data were deposited in the Sequence Read Archive (SRA) database (http://www.ncbi.nlm.nih.gov/sra/) under accession numbers: SRR4084091, SRR4084092, SRR4084095, SRR4084096, SRR4084097, SRR4084098, SRR4084099, SRR4084100, SRR4084101, SRR4084102, SRR4084093, SRR4084094.

**Table 1:** Overview of deep sequencing and data quality control.

Sample	Raw reads num	Clean reads num	Adapter sequence	Low quality	Poly A	N
AmT1-1	28,774,864	28,407,618 (98.72%)	106,791	75,473	26	1333
AmT1-2	29,564,924	29,262,394 (98.98%)	90,115	59,827	12	1311
AmT1-3	30,217,832	29,918,578 (99.01%)	84,510	65,005	13	99
AmT2-1	31,786,112	31,429,194 (98.88%)	97,180	81,265	14	0
AmT2-2	26,715,638	26,405,020 (98.84%)	90,573	63,519	12	1205
AmT2-3	28,544,942	28,238,616 (98.93%)	81,579	71,478	7	98
AmT3-1	31,163,300	30,860,010 (99.03%)	85,022	65,121	66	1436
AmT3-2	29,534,074	28,720,324 (97.24%)	100,095	306,303	31	446
AmT3-3	30,629,132	29,758,812 (97.16%)	114,989	319,668	25	478
AmCK-1	31,148,146	30,802,898 (98.89%)	88,842	83,735	27	20
AmCK-2	31,152,632	30,802,898 (98.88%)	101,983	72,783	20	81
AmCK-3	30,237,796	29,884,378 (98.83%)	109,717	65,649	5	1338

**Table 2:** Summary of mapping clean reads to the reference genome of *Apis mellifera*.

Sample	Total reads num	Unmapped reads num	Unique mapped reads num	Multiple mapped reads num	Mapping ratio
AmT1-1	27,942,952	3,369,197	24,306,993	266,762	87.94%
AmT1-2	29,045,230	3,180,318	25,597,778	267,134	89.05%
AmT1-3	29,759,616	3,202,462	26,288,338	268,816	89.24%
AmT2-1	30,908,790	3,545,971	27,066,899	295,920	88.53%
AmT2-2	26,025,206	3,098,226	22,694,714	232,266	88.10%
AmT2-3	27,965,688	2,996,833	24,701,137	267,718	89.28%
AmT3-1	29,849,508	4,091,689	25,410,587	347,232	86.29%
AmT3-2	28,575,268	3,801,677	24,469,781	303,810	86.70%
AmT3-3	29,512,734	4,059,196	25,166,090	287,448	86.25%
AmCK-1	30,540,156	3,381,653	26,848,849	309,654	88.93%
AmCK-2	30,544,824	3,221,171	26,983,983	339,670	89.45%
AmCK-3	29,636,402	3,240,646	26,010,060	385,696	89.07%

## 2 Experimental design, materials, and methods

### 2.1 Honeybee larval gut materials

*A. m. ligustica* larvae were reared following the previously described method [2]. The diet was mixed and frozen in smaller aliquots and was pre-heated to 34 °C before use. Two-day-old larvae were taken from the combs with a Chinese grafting tool and carefully transferred to the diets (10 μL). The larvae were fed once a day with 20 μL (3-day-old), 30 μL (4-day-old), 40 μL (5-day-old) and 50 μL (6-day-old) diets adding up to 150 μL diet in total.

Based on the method described by Jensen et al. [3], a fresh chalkbrood mummy was sterilized using 10% sodium hypochlorite for 10 min and then rinsed in sterile distilled water for 2 min; subsequently, the mummy was cut into smaller pieces and cultured at 25 °C on plates of Potato dextrose agar (PDA) medium, and at 7 days after culturing, fresh spores of *A. apis* were purified and used to feed 3-day-old larvae (treatment group) at a final concentration of 10^7^ spore/mL. The larvae consumed all diet were used for further study, whereas the larvae cannot consumed all diets were discarded. Three-day-old larvae in control groups were fed with an artificial diet without *A. apis* spores. Culture plates (NEXT, China) were incubated at 95% RH and 33 °C according to the method described by Aronstein et al. [4]. The honeybee larval gut is the main site *A. apis* parasitizes and larvae usually die before the prepupa stage. Twenty-one guts of 4-, 5- or 6-day-old honeybee larvae from *A. apis*-infected groups (AmT1, AmT2, and AmT3), 21 guts of 4-day-old honeybee larvae from control group (AmCK) were harvested, immediately frozen in liquid nitrogen and stored at −80 °C.

### 2.2 Total RNA isolation, cDNA library preparation and illumina sequencing

Total RNAs were extracted from each sample (pool of seven larval guts) using a TRIzol Kit (Promega, USA) according to the manufacturer's instructions. RNA quality was checked with a 2100 Bioanalyzer (Agilent Technologies, USA) and RNase-free agarose gel electrophoresis. The total RNA concentration was detected with a 2100 Bioanalyzer. RNA samples were stored at −80 °C for later library construction and next-generation sequencing. Experiments were conducted using three replicas for each sample from both *A. apis*-treated groups and control group. Totally, 12 RNA libraries were constructed, representing larval samples from *A. apis*-infected groups and control group. The libraries were as follows: AmT1-1, AmT1-2 and AmT1-3 were replicate libraries for 4-day-old larvae from the *A. apis*-treated group, AmT2-1, AmT2-2 and AmT2-3 were replicate libraries for 5-day-old larvae from the *A. apis*-treated group, AmT3-1, AmT3-2 and AmT3-3 were replicate libraries for 6-day-old larvae from the *A. apis*-treated group, and AmCK-1, AmCK-2 and AmCK-3 were replicate libraries for 4-day-old larvae from the control group.

Oligo (dTs) were used to isolate poly (A) mRNA, which was then fragmented followed by cDNA synthesis using random hexamers. Next, second-strand cDNAs were synthesized using RNase H and DNA polymerase I. The double-stranded cDNAs were purified using the QiaQuick PCR extraction kit (QIAGEN, Germany). The required fragments were purified by agarose gel electrophoresis and then enriched by PCR amplification. Finally, the amplified fragments were sequenced on the Illumina HiSeq™ 2500 platform (GeneDenovo Co., China) using 125 bp pair-end strategy. The raw data produced in this research have been deposited in the NCBI SRA Database under accession numbers: SRR4084091, SRR4084092, SRR4084095, SRR4084096, SRR4084097, SRR4084098, SRR4084099, SRR4084100, SRR4084101, SRR4084102, SRR4084093, SRR4084094.

### 2.3 Quality control and mapping of RNA-seq data

Firstly, the original sequencing image data were transferred into sequence data via base calling, which is defined as raw data or raw reads stored in the fastq format. Secondly, raw reads of all 12 samples were pre-processed by removing adaptor sequence and reads with more than 5% unknown nucleotides; low-quality reads, defined as reads where the percentage of low-quality bases of quality (*Q*) value ≤ 5 was more than 50% in a read, were also removed. Finally, the clean reads were aligned to the *A. mellifera* genome assembly Amel_4.5 (http://www.ncbi.nlm.nih.gov/genome/48?genome_assembly_id=22683) using SOAP aligner/soap2 with the threshold that no more than two mismatches were permitted in the alignment.
